# Maltol-Incorporated Acetylated Cassava Starch Films for Shelf-Life-Extension Packaging of Bakery Products

**DOI:** 10.3390/polym14245342

**Published:** 2022-12-07

**Authors:** Khwanchat Promhuad, Nattinee Bumbudsanpharoke, Kiattichai Wadaugsorn, Uruchaya Sonchaeng, Nathdanai Harnkarnsujarit

**Affiliations:** 1 Department of Packaging and Materials Technology, Faculty of Agro-Industry, Kasetsart University, 50 Ngam Wong Wan Rd., Latyao, Chatuchak, Bangkok 10900, Thailand; 2 Center for Advanced Studies for Agriculture and Food, Kasetsart University, 50 Ngam Wong Wan Rd., Latyao, Chatuchak, Bangkok 10900, Thailand

**Keywords:** food packaging, active packaging, cassava starch, advanced material, shelf-life

## Abstract

Maltol is widely used as a flavor enhancer in baked goods and has an antimicrobial function. Maltol can also be incorporated into biopolymer films to produce active biodegradable packaging for bakery products. This research investigated the incorporation of 1–10% maltol into acetylated cassava starch films as functional packaging for shelf-life extension of butter cake. Films were determined for morphology, chemical interaction and packaging properties. Infrared absorption indicated H-bonding between starch and maltol, while plasticization effects decreased mechanical relaxation temperature. Microstructures showed enhanced smoothness at up to 3% maltol, while maltol crystallization occurred at higher concentrations, giving non-homogeneous matrices. Tensile strength and elongation at break reduced by 37% and 34%, respectively, with the addition of maltol up to 10%. Maltol concentration modified the hydrophilicity and molecular mobility of the matrices, impacting water vapor and oxygen permeability. Films incorporated with maltol were used as packaging for preservative-free butter cake and delayed visible mold growth at room temperature. Starch films with maltol at 1–5% delayed fungal growth by up to 2.7–times, while films containing 10% maltol inhibited mold growth by 6–times (up to 19 days of storage). Incorporating maltol into starch films produced bioactive materials, extending shelf-life while maintaining the aroma of bakery products.

## 1. Introduction

Sustainable packaging has now become a global trend in the food industry to reduce the environmental impact of package waste. Numerous plant-derived biopolymers, including starch and other polysaccharides, have high potential to produce biodegradable films. Starch contains abundant biological macromolecules that form networks and can be converted into different packaging forms, such as films, trays and bags [1,2,3,4,5,6,7,8]. However, biopolymer-based packaging has poor barrier properties compared to conventional plastic. Improving the functional performance of biomaterials is, therefore, a research highlight to increase the protective function of food packaging [4,9,10,11,12,13]. The incorporation of active ingredients, such as inorganic nanoparticles, essential oils, food preservatives and probiotics, into starch-based materials has recently been developed as active packaging that can preserve food quality through the decontamination of spoilage and pathogens [4,7,8,11,14,15,16,17,18]. Functional packaging can efficiently extend product shelf-life, thereby reducing food loss and food waste to promote sustainability. The selection of appropriate active compounds that are compatible with food products is necessary for commercial biopolymer-based active packaging.

Maltol (3-hydroxy-2-methyl-4-pyrone) is a well-recognized flavoring agent and flavor enhancer approved by the USFDA and the European Parliament for use in and on foods, including bread, cake and other bakery products (Regulation (EC) No 1334/2008). Maltol occurs in numerous natural resources, including kojic acid, steroids and the anticoagulant warfarin, with metal chelating activity, forming a stable complex with many metallic ions for various biological treatments [19]. Recently, the antimicrobial efficacy of maltol and its synergistic effects with cationic surfactant molecules has been demonstrated against three bacterial strains, namely *Escherichia coli*, *Staphylococcus aureus* and *Pseudomonas aeruginosa,* and the two fungal strains *Candida albicans* and *Aspergillus brasiliensis*. The morphology of the microbial cells was evaluated using electron microscopy imaging, while the cell permeability assay showed efficacy to inhibit microbial growth and proliferation [20]. Maltol has been described as having a sweetened caramel-like flavor and shows potential for use as an antimicrobial substance to extend the shelf-life of foods. As a flavor enhancer, maltol is compatible with bakery products. The incorporation of maltol into biopolymers can produce antimicrobial food packaging, which can extend shelf-life.

Microbial growth, particularly, the appearance of fungal colonies on bread, cake and other bakery products, causes rejection by consumers. Numerous strategies have been developed in the bakery industry to achieve microbiological stability and retain product quality, including utilization of high-quality raw materials, lowering microbial initial load, prevention of post-process contamination, e.g., aseptic environment, and post-baking treatments, e.g., irradiation of UV and infrared light [16,21]. Active packaging incorporating volatile compounds, e.g., ethanol, cinnamaldehyde, carvacrol and oregano essential oils, has also been developed to control fungal growth in bakery products [11,16,18]. Compatibility between these volatiles and bakery products is essential to prevent food contamination by foreign aromas. Maltol has a compatible aroma with bakery products as a potential ingredient to produce active packaging. Recently, Saud et al. (2019) [22] synthesized maltol-functionalized chitosan polymers that demonstrated antimicrobial activity against *Escherichia coli*. To the best of our knowledge, no previous investigations have been conducted on maltol-incorporated active films for shelf-life extension of food products.

This research investigated maltol and starch interaction on morphology, properties and performance as functional active packaging for bakery products. Films containing different concentrations of maltol (0–10%) were investigated for morphology, chemical interaction, thermal stability, mechanical and barrier properties and application for butter cake packaging.

## 2. Materials and Methods

### 2.1. Film Preparation

Films were produced from acetylated cassava starch (AS, DS 0.01–0.03, Siam Modified Starch Co., Ltd., Pathum Thani, Thailand) mixed with maltol powder (MAL, Sigma-Aldrich Chemie GmbH, Buchs, Switzerland) at 0%, 1%, 3%, 5% and 10% w/w total solid. Glycerol (Asian Scientific Co., Ltd., Samut Prakan, Thailand) was added to the mixtures at weight ratio solid:glycerol 100:35. The mixtures were stirred at 90 °C for 60 min using a stirrer at room temperature. After homogeneous mixing, 40 g solutions (electronic balance, Sartorius BP3100 S, Sartorius Lab Instruments GmbH & Co. KG., Göttingen, Germany) were poured onto polystyrene Petri dishes (diameter 140 mm) and dried in a hot-air oven at 50 °C for 13 h. The dried films were removed from the Petri dishes and kept in a humidity-controlled space for at least 48 h at 25 ℃ and 50% relative humidity (RH).

### 2.2. Scanning Electron Microscopy (SEM)

Microstructures of the films were determined using a scanning electron microscope (FEI Quanta 450, Thermo Fisher Scientific, Waltham, MA, USA) with a 10 kV accelerating voltage. Liquid nitrogen was used to cut the films into small pieces. A small sample (10 mm × 10 mm) was placed on a metal stub and coated with gold (approximately 10 nm thickness) using a sputter coater (SPI-Module, Structure Probe, Inc., West Chester, PA, USA).

### 2.3. X-ray Diffraction (XRD)

Crystallinity of the films was determined by X-ray diffraction (Diffractometer D8, Bruker AXS, Karlsruhe, Germany). The films were scanned within 2θ range from 4° to 40° at 40 kV voltage and 40 mA current. The scanning rate was 0.8°/s with 0.02° step size.

### 2.4. Fourier-Transform Infrared Spectroscopy (FTIR)

Infrared absorption spectra of the acetylated cassava starch films were measured using attenuated total reflectance Fourier-transform infrared spectroscopy (ATR-FTIR, Tensor 27 FTIR, Bruker Optics GmbH, Aetlingen, Germany). Spectra were recorded in triplicate and averaged at a resolution of 4 cm^−1^ over 64 scanning times. Wavenumbers of 500 to 4000 cm^−1^ were used to scan the films.

### 2.5. Dynamic Mechanical Thermal Analysis (DMTA)

Stress relaxation of cassava starch films was determined using dynamic mechanical analysis (DMTA, Mettler Toledo-DMA 1, Greifensee, Switzerland). Tension-mode frequencies were 0.5, 1.0 and 5.0 Hz at −100 to 100 °C and the scanning rate was 2 °C/min.

### 2.6. Thermal Degradation Behavior of the Films

Thermogravimetric analysis (TGA) and differential scanning calorimetry (DSC) were investigated by simultaneous TGA/DSC experiments (TGA/DSC3+, Mettler Toledo, Greifensee, Switzerland) at a heating rate of 10 °C/min from 30 to 700 °C under a nitrogen atmosphere. The film sample for each test was weighed (5–10 mg) in an alumina crucible. The derivative thermogravimetry (DTG) was calculated by Mettler STARe software v10.00.

### 2.7. Mechanical Properties

Tensile strength (TS), elongation at break (EB) and Young’s modulus (YM) (ASTM D882–10, 2000) were determined using an Instron Universal Testing Machine (Model 5965, Instron, Norwood, MA, USA) with a crosshead speed of 500 mm/min. The films (150 mm × 25 mm) were stored at 50% RH for 48 h before measurement. Data were averaged from ten specimens.

### 2.8. Water Vapor Permeability

Water vapor transmission rate (WVTR) was determined by the standard cup method (ASTM E96/E96M–12). Cassava starch films (7 cm diameter circles) were placed in a metal cup containing dried silica gel and the O-ring was sealed with paraffin wax. The cups were stored in a humidity chamber (Binder KBF 720, GmbH, Tuttlingen, Germany) at 25 ± 2 °C and 50 ± 2% RH and weighed until constant weight. The WVP (g·mm·Pa^−1^·m^−2^·day^−1^) was calculated from Equation (1):

(1) $$\mathrm{WVP}\;=\left(\mathrm{WVTR}\;\times \;L\right)/\Delta P$$

where water vapor transmission rate (WVTR, g·m^−2^·day^−1^) derives from the slope of the linear weight increase, L (mm) is the thickness using a thickness gauge (Mitutoyo Manufacturing Company, Kanagawa, Japan) and ΔP (Pa) is the water vapor pressure difference between two specific surfaces of film, under specified temperature and humidity conditions.

### 2.9. Oxygen Permeability

Oxygen transmission rate (OTR) was determined using an oxygen permeation analyzer (Model 8501, Illinois Instruments, Johnsburg, IL, USA) following ASTM D3985. Cassava starch films (7 cm diameter circles) were conditioned at 25 °C and 50% RH for 48 h. Oxygen permeability (OP; cm³·mm·m^−2^·day^−1^·atm^−1^) was calculated from Equation (2):

(2) $$\mathrm{OP}\;=\left(\mathrm{OTR}\;\times \;L\right)/\Delta P$$

where ΔP (atm) is the difference in partial pressure between each side of the films which was determined to be 1 atm and L is the thickness (mm). The OP data were averaged from three specimens.

### 2.10. Application as Active Packaging for Bakery Products

Butter cake without preservatives was purchased from a local bakery in Bangkok, Thailand, kept at room temperature and used for measurement on the same day of baking. The films were applied directly to the surface of the butter cake. The tray was also sealed with cassava starch films. Triplicate samples were stored at room temperature and visually inspected every day until fungal growth.

### 2.11. Statistical Analysis

Analysis of variance (ANOVA) at 95% was used to examine significant differences in film properties using SPSS 17 Statistics software (SPSS Inc., Chicago, IL, USA). Duncan’s multiple range test was performed to indicate significant differences among blends.

## 3. Results and Discussion

### 3.1. Microstructures 

**Figure 1:**
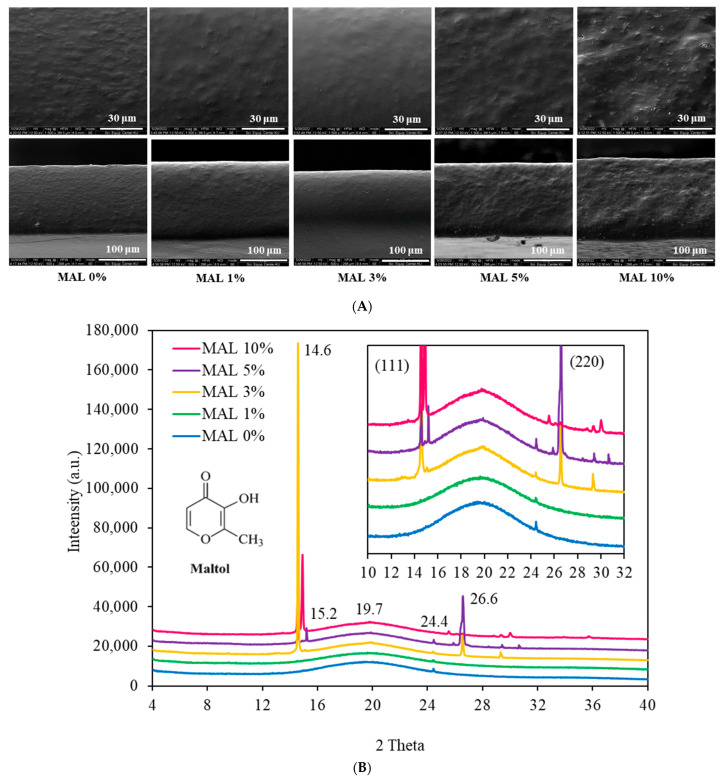
(**A**) Microstructures on surface (**upper row**) and cross-section (**lower row**) and (**B**) X-ray diffractogram of starch films containing maltol (MAL) at 0, 1, 3, 5 and 10%.

Microstructures of starch films containing different concentrations of maltol (MAL) are shown in Figure 1A. Control films (no MAL) had fine embedded granules giving a rough surface and cross-section structures that reflected the crystallites or intact starch granules in the films. Shaikh et al. (2019) [3] also found a rough film structure with several imperfections and ridges. Adding MAL up to 3% reduced the roughness and gave smoother structures, particularly in MAL 3%. Results suggested that MAL enhanced the disruption of starch granules. Conversely, increasing MAL above 3% (5% and 10%) gave a rougher surface. High MAL contents accelerated self-adhesion of MAL molecules and crystallization occurred, causing phase separation of fine crystalline particles and non-homogeneous structures, while detachment of crystallites on the surface caused fine cavities in MAL 10%, concurrent with the highest roughness. Cross-section structures were similar to the surface, with MAL 3% and 10% showing the highest smoothness and roughness, respectively. 

### 3.2. X-ray Diffraction 

X-ray diffractograms (Figure 1B) of MAL 0% showed smooth curves, typical of amorphous components. A small peak at distances between the planes of the crystals d = 3.6 Å corresponding with diffraction angle at 24.4° was due to the cassava starch component [14]. However, this small diffraction peak (2θ = 24.4°) was eliminated in MAL 10%, suggesting that incorporating 10% maltol eliminated starch crystals. Therefore, coarse particles on the film surface (SEM images in Figure 1A) were not due to starch crystallites but solely attributed to maltol clumps. Adding MAL from 3% to 10% gave sharp diffraction peaks at 2θ = 14.6° (111), 14.9° (111) and 26.6° (220), attributed to MAL crystals [22]. Burgess et al. (1996) [23] indicated two polymorphs of maltol (C_6_H_6_O_3_) crystals as (i) near planar chain of maltol connected by H-bonding and (ii) molecules with mutually hydrogen-bonded dimers. The presence of a weaker R-C-H---O-R’ bond stabilized the chains in (i) and connected dimers in (ii) [23]. The intensity of these diffraction peaks varied depending on MAL concentrations, suggesting different lattice structures. Maltol has two donor sites, namely carbonyl oxygen (C=O) and hydroxyl (O-H) [19]. A sharper peak at 2θ = 19.7° was found in films containing MAL at 3% and higher (Figure 1B). These active sites modified bonding between maltol–maltol and maltol–starch, which subsequently influenced the structure of the crystalline lattice. 

### 3.3. FTIR

**Figure 2:**
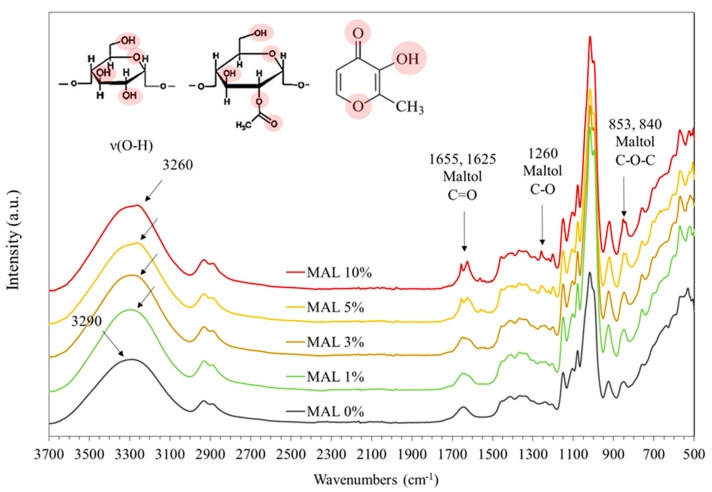
FTIR absorption spectra of starch films containing maltol (MAL) at 0, 1, 3, 5 and 10%.

The IR absorption spectra of films containing different concentrations of maltol are shown in Figure 2. The fingerprint region between 500 and 1500 cm^−1^ showed the typical absorption peaks of starch and maltol components. FTIR peaks ranging from 800 to 1200 cm^−1^ were attributed to stretching vibrations of C-O and C-C bonding of maltol and the glycosidic bonds of starch [24]. Maltol showed absorption stretching vibration peaks at 840, 853 (C-O-C), 920 (C-O-H), 1260 (C-O) and 1560 (C=C) cm^−1^ [19,22], and the magnitudes of these peaks increased with increasing maltol contents. The peak at 925 cm^−1^, indicating C-O stretching in starch (MAL 0%), shifted to lower wavenumbers when adding maltol (921 cm^−1^ for MAL 5%), reflecting modified C-O stretching (C-O-H and C-O-C) in starch molecules due to maltol. 

Modifying acetic anhydride into starch structures substitutes free hydroxyl groups on glucose units with acetyl groups that are hydrophobic [3]. Neat acetylated starch film (MAL 0%) showed absorption between 1600 and 1700 cm^−1^, indicating carboxylate groups of acetylated modified cassava starch (Figure 2). Adding maltol above 3% caused a distinct peak in this region. Absorption peaks at 1625 and 1655 cm^−1^ were ascribed for C=O stretching vibration [19,25]. Maltol contains C=O carbonyl groups with two polymorphic crystalline structures [23]. These distinct peaks likely reflected the two different polymorphs of maltol crystallites, with diverse vibration energy of the C=O bonding. 

Starch films (MAL 0%) had a wide absorption peak between 3000 and 3700 cm^−1^, attributed to O-H stretching vibration centered at 3290 cm^−1^. This absorption peak was sensitive to inter- and intra-molecular hydrogen bonding. Incorporation of maltol shifted the peak to lower wavenumbers and intensified the magnitude of the stretching vibration peak at 3260 cm^−1^. The aforementioned red shift in IR wavenumbers suggested increasing bond length and decreasing force constant [26]. Interaction between starch and maltol via the hydroxyl groups of both molecules formed intermolecular hydrogen bonding and elongation of H-bonding in starch molecules, which absorbed IR at lower wavenumbers.

### 3.4. DMTA

**Figure 3:**
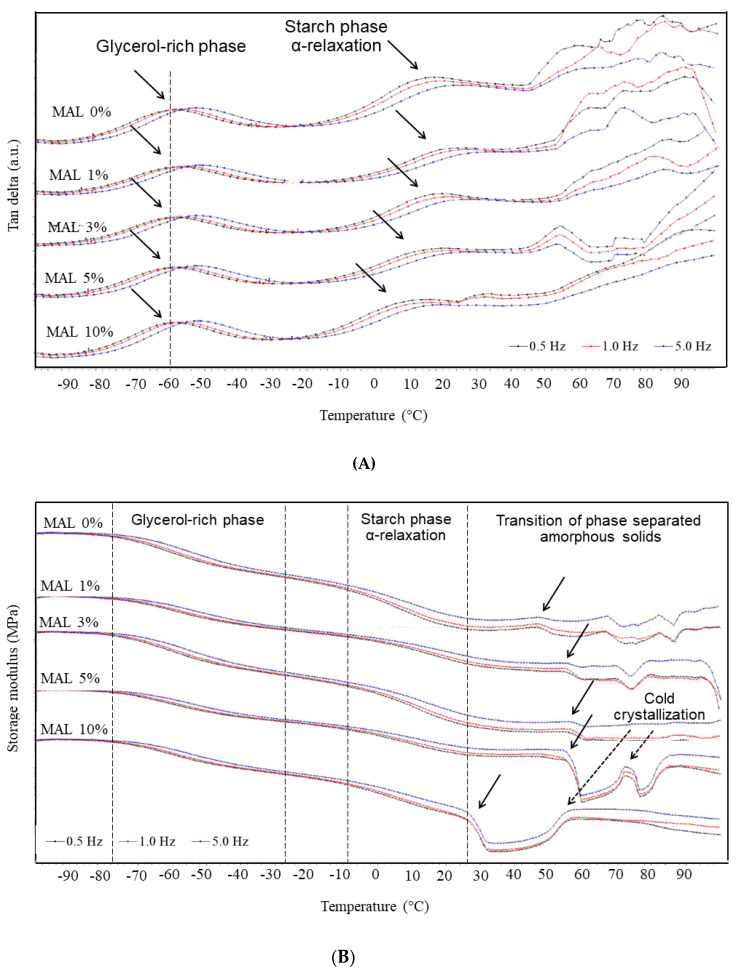
DMTA (**A**) tan delta and (**B**) storage modulus of starch films containing maltol (MAL) at 0, 1, 3, 5 and 10%.

DMTA analysis of the films at different frequencies (0.5, 1.0 and 5.0 Hz) showed peaks of tan δ, which are frequency dependent, indicating mechanical relaxation temperature (Figure 3A). Two distinct peaks of tan δ are commonly observed in starch-based films at −80 to −30 °C and 20 to 50 °C. The peak of tan δ at higher temperature indicated α-relaxation temperature (Tα) of the starch polymers as a result of glass transition from amorphous glassy to rubbery state [27], while the peak at lower temperature reflected relaxation behavior of the glycerol-rich phase in the film matrices [14,28,29]. Addition of maltol showed insignificant effects on the relaxation temperature at lower temperature transition. Conversely, Tα decreased with increasing maltol concentrations, indicating the plasticization effects of maltol on starch molecules. Forssell et al. (1997) [28] also demonstrated a shift toward lower relaxation temperature in the starch-rich phase due to plasticization effects, while relaxation temperature of the glycerol-rich phase only decreased with increasing water content (independent of glycerol content). Water is the strongest plasticizer for hydrophilic materials. Results in Figure 3A suggested that interaction between maltol and glycerol insignificantly depressed the phase transition temperature in the glycerol-rich phase. Conversely, FTIR indicated H-bonding between starch and maltol, which effectively plasticized the starch-rich phase and decreased glass transition temperature (T_g_), giving reduced Tα. 

The drop-in storage modulus suggested phase transition of the amorphous polymer components [27,30]. MAL 0% films showed two major drops in storage modulus due to phase transitions of the glycerol-rich phase and starch polymers (Figure 3B). Moreover, several small transitions at 50 °C and above were found due to transitions of dispersed particulate components, as shown in the SEM images (Figure 1A). The transition above 50 °C decreased with increasing maltol to 3%, corresponding with a more homogeneous structure and elimination of the dispersed particles in the film matrices. A sharp drop in storage modulus was found in MAL 5% and MAL 10%, suggesting the transition temperature of maltol clumps. Results suggested that maltol formed amorphous glass (maltol-rich phase) at low temperature, which was phase separated from the starch matrices. 

Moreover, MAL 3% and 5% showed increased storage modulus, suggesting cold crystallization of film components [27]. As maltol contents increased from 5 to 10%, the cold crystallization peak in MAL films became more obvious. The magnitude was also stronger and occurred at a lower temperature in high maltol contents (MAL 10%). Accordingly, results indicated that maltol induced crystallization in the system.

### 3.5. Thermal Degradation Behavior of the Films

**Figure 4:**
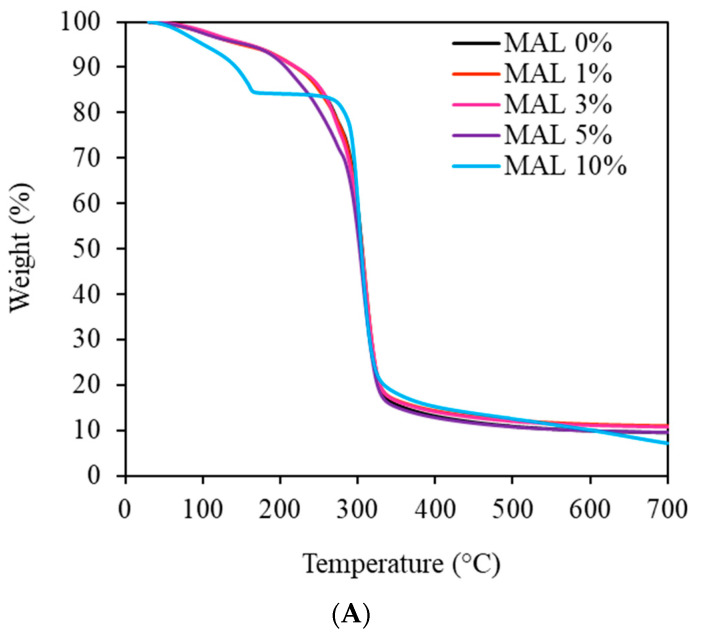

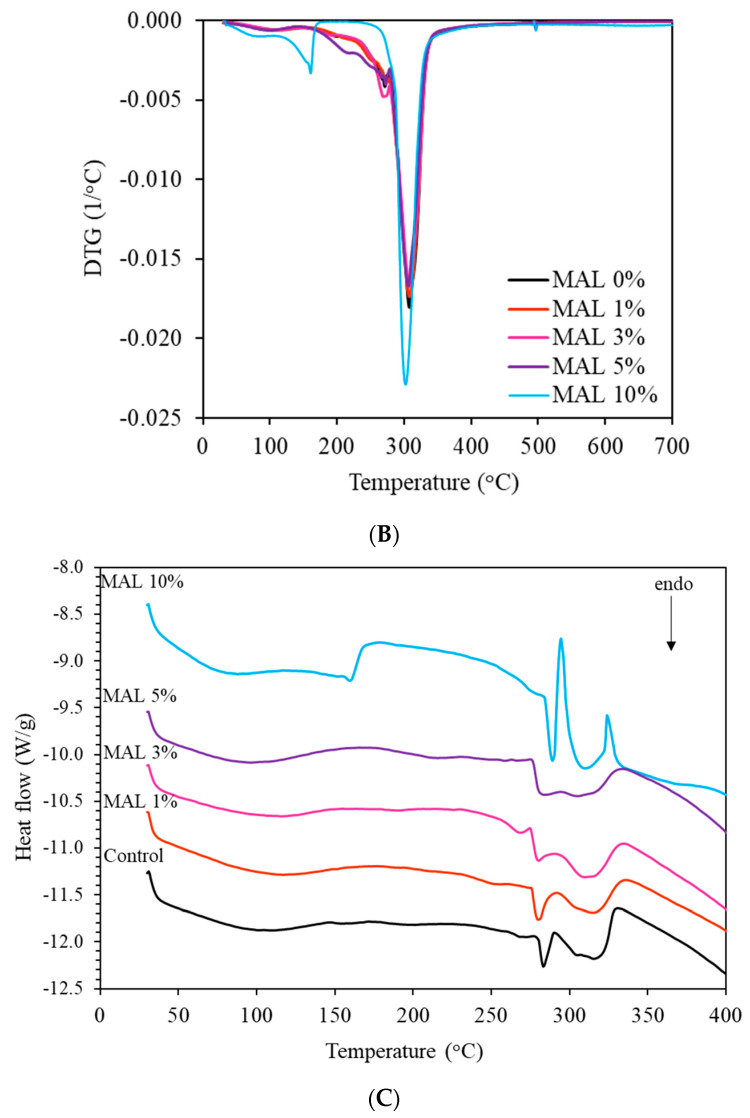
Thermal degradation behavior as (**A**) weight loss, (**B**) first derivative weight loss and (**C**) DSC thermograms of starch films containing maltol (MAL) at 0, 1, 3, 5 and 10%.

Thermal degradation of the films as weight loss and 1st derivative weight loss are shown in Figure 4A,B. Films with maltol 1–3% had a weight reduction step of approximately 8%, starting at around 100 °C (Figure 4A). A minor degradation starting at 190 °C was followed by a sharp weight loss at 240 °C, attributed to volatilization of glycerol and starch components, respectively [28,31]. Adding maltol up to 3% gave identical thermal degradation behaviors. Conversely, higher amounts of maltol (5 and 10%) showed destabilization of starch films. A sharp weight loss of 18% at 160 °C was found in MAL 10%, followed by a sharp weight loss at 280 °C. 

Figure 4B shows steps of thermal degradation in starch films (MAL 0%). Water evaporation peaked at around 100 °C, followed by glycerol volatilization at 190 °C. A sharp peak at 310 °C, attributed to degradation of starch molecules, merged with a peak at 280 °C, attributed to major native starch and partially substituted acetylated starch, respectively. Zhang et al. (2009) [32] indicated less stability in partially substituted acetylated starch (degree of substitution ranging from 0.09 to 1.51) than native, giving degradation at a lower temperature. MAL 1% to 3% had identical thermal degradation behavior. Conversely, MAL 10% films showed diverse degradation behavior. The magnitude of water evaporation was highest, reflecting the highest water absorption and evaporation. A second peak maximum at 160 °C had higher magnitude than the other samples, suggesting volatilization of glycerol at lower temperature. Degradation of native and partially modified starch components in MAL 10% merged and formed a sharp degradation peak at a lower temperature than the others. 

Figure 4C shows phase transition temperature, as determined by DSC. MAL 0% had a sharp endothermic peak at 280 °C followed by a distinct peak above 290 °C. The peak temperature at 280 °C decreased when adding maltol up to 5%, corresponding with a merge of the peak above 290 °C. Results suggested that maltol destabilized the starch phase due to decreasing portions of crystalline starch. However, MAL 10% showed two distinct sharp peaks, reflecting the homogeneous structures of starch components. Destabilization of glycerol in MAL 10% films eliminated interaction between starch and glycerol, giving more homogeneous starch structures. A sharper peak at high maltol content indicated higher amounts of crystallites, requiring higher energy to break the lattice bonds, which commonly have denser structures [31,33].

### 3.6. Mechanical Properties

**Figure 5:**
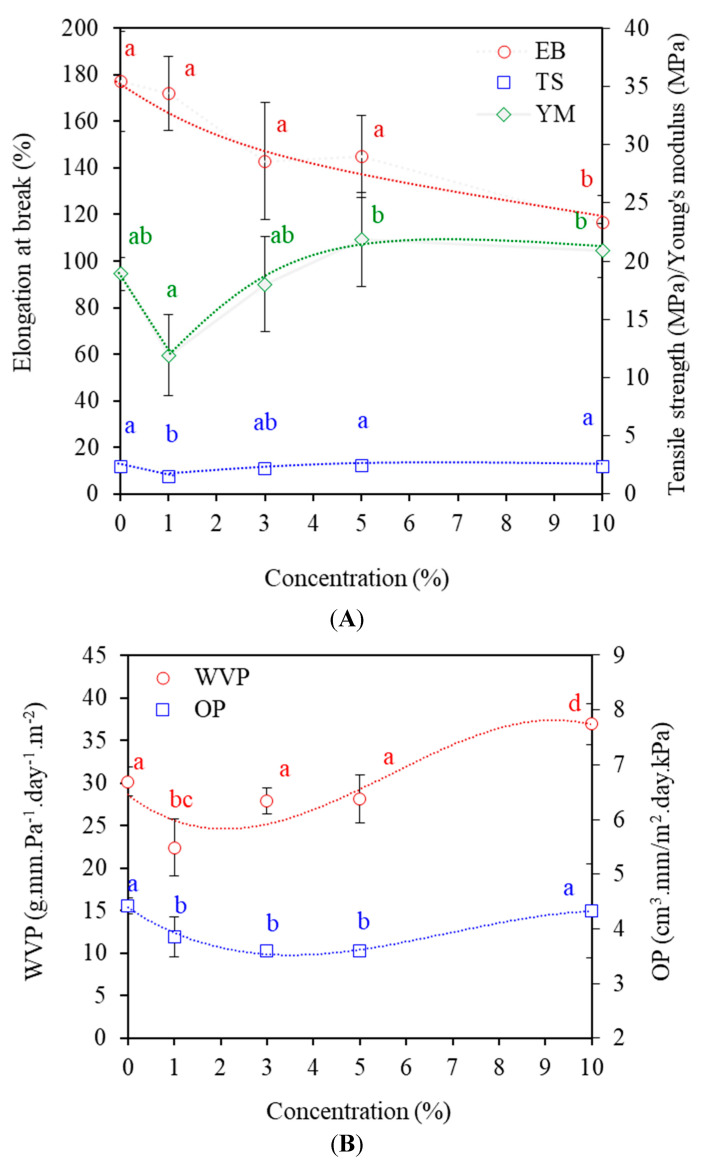
(**A**) Tensile properties as tensile strength (TS), elongation at break (EB) and Young’s modulus (YM) and (**B**) barrier properties as water vapor permeability (WVP) and oxygen permeability (OP) of starch films containing maltol (MAL) at 0, 1, 3, 5 and 10%. Different letters (a, b and c) indicate significant difference (*p* ≤ 0.05) between maltol concentrations.

Tensile properties of MAL films as tensile strength (TS) and elongation at break (EB) are shown in Figure 5A. TS reflects the ability of a biopolymer film to withstand maximum applied stress without breakage [14]. TS decreased by 37% with the addition of maltol at 1%, possibly due to incorporated maltol and, hence, non-homogeneity of the polymer matrices. Less homogeneous networks cause fewer areas for stress distribution against external forces, reducing the ability to bear external loads. However, increasing maltol to 3% improved TS, corresponding to the elimination of dispersed starch granules and giving homogeneous structures (Figure 1A). Further increasing maltol to 10% insignificantly affected TS, although SEM images indicated non-homogeneous structures due to dispersion of maltol crystallites. Tensile properties of polymers vary depending on several factors, including microstructures (e.g., homogeneity, porous-dense, micro-voids and crack), morphology (amorphous–crystalline fraction, molecular orientation of polymer molecules) and interaction between blend components (bonding, adhesion) and plasticization [1,4,5,13,14]. Our results suggested that strong interaction between the surfaces of maltol crystallites and starch networks improved the network distribution of stress and strength, effectively compensating for the non-homogeneity of the matrices.

Figure 5A shows that maltol had insignificant effects on EB up to 5%, while 10% maltol significantly decreased EB values (*p* ≤ 0.05) by 34%. DMTA analysis indicated the plasticization effects of maltol in starch films, potentially improving flexibility and elongation. However, less homogeneous structures due to the dispersion of maltol clumps reduced extensibility. Higher crystallinity in films causes brittleness, with less flexible structures and lower stretchability [14]. YM decreased by 37% with 1% maltol, as similarly found in TS, while YM increased with increasing maltol above 3%, indicating increased stiffness. Rigid maltol particles reinforced the polymer matrices and reduced extensibility, thereby improving the rigidity of the matrices [17].

### 3.7. Barrier Properties

Film permeability controls the shelf-life of packaged foods. Water vapor permeability (WVP) and oxygen permeability (OP) values are shown in Figure 5B. Adding 1% maltol decreased WVP (*p* ≤ 0.05) by 26% due to decreasing numbers of starch granules and more homogeneous matrices that limited diffusion of water vapor. However, further increasing maltol increased WVP. Maltol interacted with starch via H-bonding and plasticized the matrices. Moreover, maltol has a hydrophilic component that absorbed water and increased water plasticization [5]. Increasing hydrophilicity in the matrices increases WVP [1,8].

OP values significantly decreased (*p* ≤ 0.05) by 19% when adding maltol up to 5% (Figure 5B). Diatomic oxygen molecules (O_2_) share a lone pair electron, having equal electronegativity and being non-polar. Maltol addition improved the hydrophilicity of the films by preventing diffusion of non-polar oxygen. Kishi et al. (2020) [34] also indicated increasing OP values with increasing hydrophobicity of biopolymer films. The transport phenomena of oxygen are well described by matrix hydrophobicity, which modified the absorption and affinity of transport molecules. Less affinity between hydrophilic matrices and non-polar molecules reduced absorption of the molecules at the interface and retarded diffusion, giving lower permeability. Increasing maltol to 10% improved OP due to the plasticization effects of film matrices, which increased free volume (molecular distance) and improved molecular mobility and diffusion rates of volatile compounds through solid amorphous matrices [6,35,36]. Results indicated that transport phenomena in maltol-plasticized starch films were dependent on concentrations that modified the hydrophilicity and molecular mobility of the matrices.

### 3.8. Application as Active Packaging for Bakery Products 

**Figure 6:**
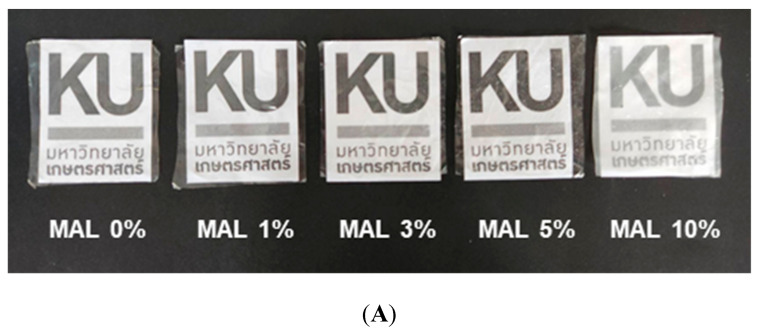

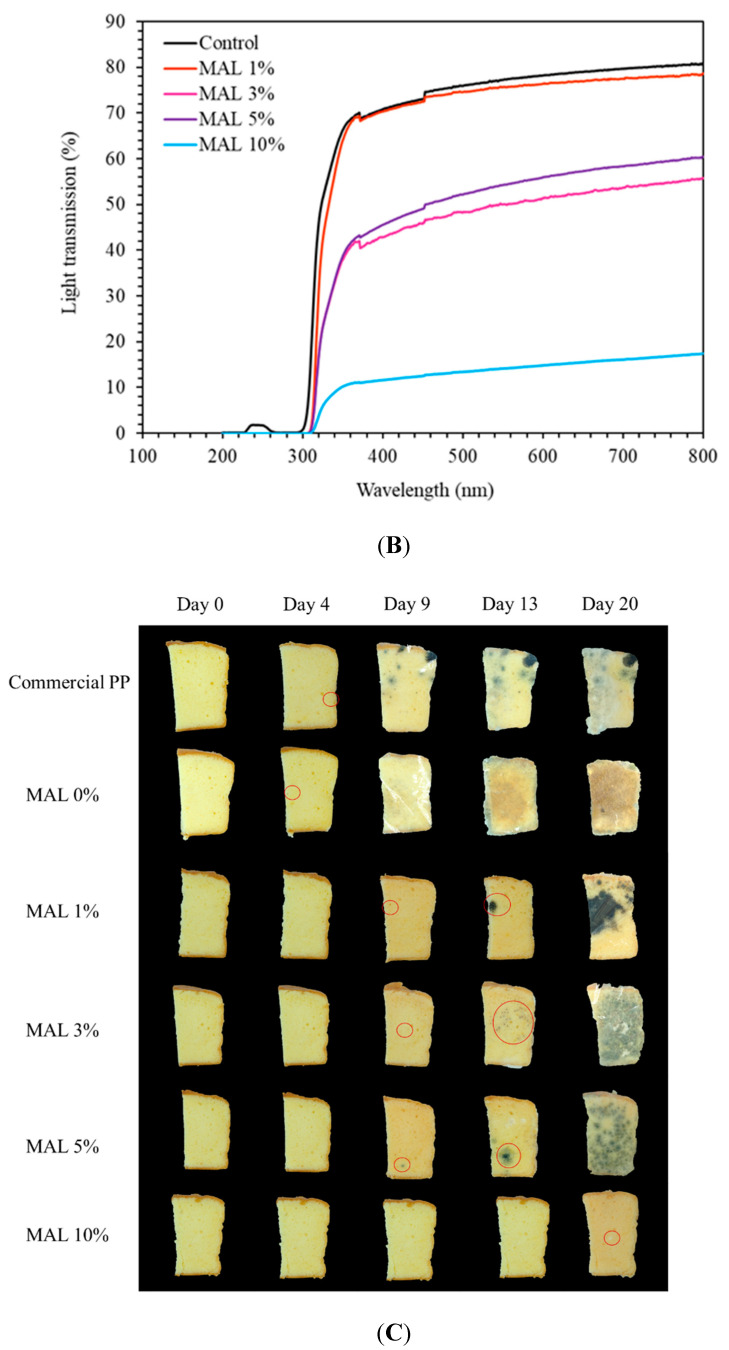
(**A**) Film appearance, (**B**) light transmission and (**C**) mold growth in cake packaged in starch films containing maltol (MAL) at 0, 1, 3, 5 and 10%. Red circles indicate visible mold growth.

Figure 6A shows the appearance of films with different maltol concentrations. Higher maltol contents gave a turbid film character due to crystal clumps of maltol that impacted clarity. Light transmission of the films reduced by 1.5 times when adding 3% and 5% maltol (Figure 6B). MAL 10% gave the lowest light transmission at less than 15% in the visible region due to the high amount of dispersed maltol clumps (Figure 1A). 

Maltol is a flavor enhancer with antimicrobial functions and a sweet caramel-like aroma compatible with bakery products [19,20]. Starch films containing maltol were determined for functional packaging of butter cake (Figure 6C). Butter cake packaged in commercial PP and MAL 0% films had visible mold growth on day 4 of storage. Adding maltol to the films from 1 to 5% effectively delayed mold growth by up to 8 days, while MAL 10% was the most effective and prevented mold growth at up to 19 days of storage. MAL 0% and MAL 10% had identical OP (Figure 5B), while MAL 10% effectively delayed mold growth, confirming that incorporating maltol had a significant effect on antifungal properties. 

Morassi et al. (2018) [21] found that the most abundant fungal strains in cake emanated from the raw materials and the processing air environment as *Penicillium*, *Aspergillus*, *Cladosporium* and *Aspergillus* with sexual state *Eurotium*, respectively. Maltol is a volatile compound and release of maltol from MAL films and further absorption onto the surface of bakery products modified fungal activity and effectively prevented growth. Ziklo et al. (2021) [20] demonstrated antifungal (*Candida albicans* and *Aspergillus brasiliensis*) and antibacterial (*Escherichia coli*, *Staphylococcus aureus* and *Pseudomonas aeruginosa*) efficacy of maltol, including enhancing potassium leakage from microbial cells, over-folding of cell membranes and disintegration of regular cell structures. Maltol also caused severe disintegration of fungal cell walls, forming distance between intracellular components and receding cell membrane, and consequent lysis of cell organelles and intracellular components [20]. Results indicated the high efficacy of films containing maltol as active packaging to extend the shelf-life of bakery products.

## 4. Conclusions

Incorporation of maltol into starch films at 1–10% produced functional active packaging that effectively delayed fungal growth and extended the shelf-life of butter cake. Maltol formed H-bonding with starch and plasticized the matrices, thereby decreasing the relaxation temperature. Starch granules were eliminated with the addition of maltol; however, less homogeneous film microstructures were formed at higher maltol contents above 3%. Degree of crystallinity of the films improved when adding maltol at 3% and above because maltol induced crystallization. Mechanical properties of the films were influenced by addition of maltol at up to 10%. Permeability in starch films containing maltol was dependent on concentration because maltol modified the hydrophilicity and molecular mobility of the matrices. Adding 1–5% maltol delayed fungal growth by 2.7 times. Films with 10% maltol delayed visible mold growth by approximately 6–times (up to 19 days of storage) because release of maltol displayed antifungal properties. Incorporating maltol with starch matrices as packaging film effectively extended the shelf-life of bakery products. Antimicrobial tests should be quantitative and sensory quality assessment for consumer perception should be carried out for further investigation.

## Data Availability

Not applicable.
